# Population Size and Migration of *Anopheles gambiae* in the Bancoumana Region of Mali and Their Significance for Efficient Vector Control

**DOI:** 10.1371/journal.pone.0010270

**Published:** 2010-04-21

**Authors:** Ibrahima Baber, Moussa Keita, Nafomon Sogoba, Mamadou Konate, M'Bouye Diallo, Seydou Doumbia, Sékou F. Traoré, José M. C. Ribeiro, Nicholas C. Manoukis

**Affiliations:** 1 Malaria Research and Training Center, Faculté de Médicine, de Pharmacie et d'Odonto-Stomatologie, Université du Mali, Bamako, Mali; 2 Section of Vector Biology, Laboratory of Malaria and Vector Research, National Institute of Allergy and Infectious Diseases, National Institutes of Health, Bethesda, Maryland, United States of America; University of California, Berkeley, United States of America

## Abstract

We present results of two intensive mark-release-recapture surveys conducted during the wet and dry seasons of 2008 in the villages of Fourda and Kenieroba, Mali. The former is a small fishing village by the Niger River with a moderate to high densities of *Anopheles gambiae* Giles s.s. (Diptera: Culicidae) throughout the year, while the latter is a large agricultural community 2 km inland that experiences strong seasonal fluctuation in *An. gambiae* densities. We estimate the population size of female *An. gambiae* in Fourda to be in less than 3,000 during the dry season. We found evidence of large population size and migration from Fourda in Kenieroba during the wet season, but very low numbers and no sign of migrants during the dry season. We suggest that malaria vector control measures aimed at adult mosquitoes might be made more efficient in this region and other seasonal riparian habitats by targeting disruption of mosquito populations by the river during the dry season. This would decrease the size of an already small population, and would be likely to delay the explosive growth in vector numbers in the larger inland villages as rainfall increases.

## Introduction

The burden of malaria in sub-Saharan Africa remains enormous; 90% of the estimated one million deaths from the disease per year occur there [1], [2]. While global investment in measures to reduce transmission have increased significantly in recent years [3], there is still a need to identify ways to improve the outcome of interventions against malaria vectors, because resources and capacities in the countries most affected by malaria are limited. Significant improvement in the efficiency of measures aimed at reducing malaria vector numbers can come from a more complete understanding of vector population dynamics [4], [5], especially dispersal or migration patterns [6], [7].

Yearly change in malaria vector population size is evident in many parts of the world and has been the focus of extensive research, especially in biomes of sub-Saharan Africa that have strong seasonal climate variability, such as the Sudan savannah [8]. During the dry season in these areas, population sizes of *Anopheles gambiae* can become quite small, or the vector may disappear locally [9]–[11], presenting an opportunity to improve the longevity and effectiveness of an intervention; however, the advantage of intervening at a moment of small population size will be especially affected by whether the target population of mosquitoes is contained or if it receives migrants. Particularly relevant to vector control are source-sink type dynamics [12] that should be explicitly considered when designing dry-season interventions. In this paper, we use the term “migration” with respect to individual movement over space as distinct from “dispersal” because 1) we are not focusing especially on stage specific movement and 2) we are referring to actual movement of individual mosquitoes, not to a distributional outcome of that movement at a population level (i.e. we are using the term *sensu* [13]).

Mosquito populations may exhibit source-sink metapopulation structure in some riparian areas of the Niger River, where small fishing villages are found paired with larger agricultural settlements a few kilometers inland [14]. This hypothesis holds that source populations near the river are persistent (sources) while larger inland villages experience negative growth rates for a portion of the year (sinks; [12]). It has been supported by monthly surveys showing a strong reduction in population size within a large inland village (Bancoumana) during the dry season but stable numbers in the adjoining small fishing village (Bozokin). The persistence of *An. gambiae* near the river appears to result from larval habitat production as the Niger recedes (a situation documented for other rivers also; see [15]): water pools are seen in the sandy river bed during the dry season, and these maintain larval productivity. Similar factors may explain concordant results recently reported from The Gambia [16].

In this study, we estimated the population size of *An. gambiae* in the small fishing village of Fourda during the dry and wet seasons, and its connectivity by migration to the much larger village of Kenieroba via mark-release-recapture (MRR) techniques. Further confirmation that source-sink dynamics exist in riparian areas of Mali would support interventions focused on the fishing villages near the Niger river during the dry season. Such efforts might reduce the number of mosquitoes colonizing inland areas once the rains arrive, reducing effective vector densities and shortening the transmission season inland.

## Methods

### Ethical Statement

Ethical clearance for pyrethrum spray and human landing catches (HLC) was obtained from the Institutional Review Board of the Faculty of Medicine, Pharmacy and Odonto-Stomatology, University of Bamako (Lettre no41/FMPOS du 07-06-07). HLC collectors were volunteers (18–30 years old) from the study villages, and an individual consent form was presented to them for approval before the collection. In this consent form an explanation of the study objectives and risks related to HLC collection were given, as well as the option for them to participate or not. The consent form was either read by the volunteer (for those who were literate) or read aloud to them (for those who were illiterate) by one of the research team members. The name and contact information for the lead field researcher or the PI of the project at MRTC was provided to each volunteer in case of emergency. After approval, each collector was asked to sign (if literate) or to place a finger print on the consent form. Volunteers were protected with an appropriate antimalarial prophylaxis.

The insecticide used to spray the houses was pyrethrum based, sold under the label of Premium Killer(r) (NIRA BVBA, Antwerp, Belgium), a brand commonly used by the population in this area. This product has a weak persistence, has no human toxicity under normal conditions of use, and is intended for use as an indoor spray. No house was sprayed without the approval of the owner. During the spray all meals and liquid was taken out and the owner was advised to keep the door open after collection for at least 30 min before re-entering the house. All the documents generated during the study and containing individual information are kept in a secure location at the MRTC, University of Bamako, with the identification code.

### Study Site

We conducted our study in the fishing village of Fourda, adjacent to the Niger river (12.09° N, 8.34° W), and the larger agricultural village of Kenieroba, directly inland from Fourda (12.11° N, 8.33° W). These villages are located about 71 km south-west of Bamako and about 14 km from Bancoumana, where various entomologic and epidemiologic studies focused on malaria have been conducted recently [8], [14], [17], [18]. A flood plain about 2 km wide separates Kenieroba and Fourda; it is used for flooded rice agriculture during the rainy season and vegetable cropping during the dry season.

The rainy season is from May to November and the dry season from December to April, with annual rainfall between 750 and 1500 mm. Nearly all the rainfall occurs during the rainy season. Kenieroba has 2,065 inhabitants in 690 houses, and Fourda has 275 inhabitants in about 40 houses. Individual houses are arranged in compounds of between one and two dozen houses, as is traditional in this region.

During a previous study on larval and mosquito density in this area, all potential breeding sites around Kenieroba were surveyed and georeferenced using GPS. Most of the larval habitats in Kenieroba were man made and found to dry out during the dry season. By contrast, the dry season in Fourda brings naturally occurring sandy water pools in the river bed as the water level drops, which are productive larval habitat in close proximity to the human habitations.

### Mosquito collection, marking and release

We performed daily MRR in Fourda for 3 days in March 2008 (the dry season period when *An. gambiae* population size is low) and 5 days in July 2008 (rainy season, and a period of higher mosquito numbers). Recaptures only were also conducted for an additional 4 days in March and 3 in July. The variation in protocol is due to the vastly different mosquito densities between the two periods: low densities in March allowed and required less marking. We conducted recaptures until the number of marked mosquitoes dropped to low levels. The last capture both times was conducted by pyrethrum spray catch (PSC) rather than mouth aspiration, following the protocol described in Diuk-Wasser *et al*. [19] to maximize the proportion of the existing population sampled.

During both study periods, our team of 20 workers performed mosquito collection using mouth aspirators for consecutive days from 08:00h to 13:00h in all houses in Fourda. We recorded the number of mosquitoes collected (females and males) in each structure, with details including name of the house owner, house type (thatch or metal roof), and number of sleepers. We kept collected mosquitoes in two large cages (about 4 L in volume) within a house, supplied with sugar solution in cotton and covered with a damp cloth.

Around 15:00h, we transferred mosquitoes from the large cages in batches of 25 individuals to small paper cups lined with fluorescent powder (Day-Glo Color Co., Cleveland OH, USA). A different color was used for each marking day. We recorded the number of males and females per cup. By flying and landing in the cup, mosquitoes were marked with a fine dusting of the fluorescent powder. To ensure 100% marking, we scraped cotton with powder over the net covering the cup.

We released marked mosquitoes around sunset (19:00h) at the edge of the village of Fourda. We removed the net covering the cups containing marked mosquitoes and allowed five minutes for them to disperse. Any individuals still in the cups after this time were subtracted from the estimate of the number released.

Each release experiment was followed by daily collections in all houses of Fourda. We checked all collected mosquitoes with an ultraviolet light for signs of marking; any that were found with powder were preserved in 80% ethanol. We processed all other, non-marked, mosquitoes with a different dye and released them at sunset as above.In addition to mosquito collections in Fourda, we searched for marked mosquitoes in the larger inland village of Kenieroba in both March and July.

### Taxonomic Identification

We performed PCR-based characterization of species [20] and molecular form for *An. gambiae* s.s. [21], [22] on separate samples of mosquitoes from Fourda and Kenieroba in March and July, 2008 (Table 1). Samples sizes from Fourda were quite small in March (11 mosquitoes total), so we also typed samples from the village of Bozokin, another small fishing hamlet by the river about 15 km from Fourda with similar ecologic and demographic characteristics.

**Table 1:** Species and Molecular Form Composition in Fourda, Bozokin, and Kenieroba.a,b

	March 2008				July 2008			
Village	M	S	R	H	M	S	R	H
Bozokin	20	10	4	2	19	0	0	3
Fourda	4	3	3	1	28	4	0	8
Kenieroba	45	9	7	10	57	48	1	10

a Number of individuals of each type from samples collected in March and July 2008.

b M, M molecular form; S, S molecular form; R, *Anopheles arabiensis*; H, M-S molecular form hybrids.

### Methods to Estimate Population Size

We calculated “instantaneous” estimates of the population size in Fourda using the Lincoln index with small recapture size correction [23], where we approximate the key assumptions of this method by using release and recapture data only for consecutive days. This minimizes the effects of mortality and recruitment on our estimates. The equations used are given below:
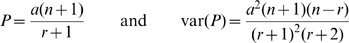



where *P*  =  population size estimate, *a*  =  number marked/released the previous day, *n*  =  number captured during the current day, and *r*  =  number captured that were marked (recaptures) the previous day.

In addition, we conducted calculations of population size based on multiple recaptures using two methods: the Fisher-Ford multiple recapture method [23] and the joint hypergeometric maximum likelihood estimator (JHE) [24] as implemented in the computer program NOREMARK [25]. The former provides allows comparison to previous work, e.g. [26], [27], while the latter is useful in providing a single estimate with confidence intervals.

Both the Fisher-Ford and JHE multiple recapture methods utilize information from multiple releases prior to the capture date (Fisher-Ford) or for the entire duration of the experiment (JHE). As recaptures are spread out over various days, these methods require a value for daily survivorship to estimate how many marked mosquitoes remain available to be caught. We made an attempt to estimate daily survivorship in both March and July, but did not use the same method both times due to differences in the numbers of mosquitoes caught each time: In March, we conducted a night collection in Fourda during the experiment to measure parity rates, which allows an estimate of survivorship [23], [28]. The number of mosquitoes captured was higher in July, so we set up separate 250 ml paper cups containing 10 marked and 10 unmarked females per collection day in Fourda to estimate survivorship and to test for any effect of the fluorescent powder on longevity. Both these methods for estimating daily survival are of limited value, so in the analysis of our data we varied survivorship to explore the variation this might induce in the estimates of population size.

## Results

### Data Summary

We captured a total of 905 females and 703 males in Fourda in March; 265 females and 107 males were successfully marked and released over the first three of seven collection days. Total recaptures were 28 females (10.6% of all the marked females) and 4 males (3.7% of all the marked males). In July, total captures in Fourda were 2185 females and 1514 males, of which 874 females and 326 males were successfully marked and released over the first five of eight collection days. Total recaptures in July were 31 females (3.5% of all the marked females) and 6 males (1.8% of all the marked males).

**Figure 1:**
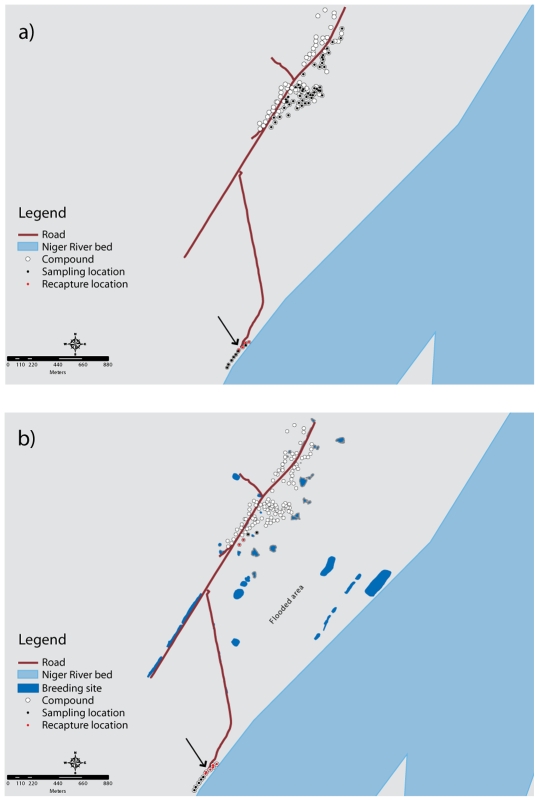
Sampling and recapture locations in Fourda and Kenieroba with breeding sites. Maps show Fourda (to the South) and Kenieroba (North) in March (a) and July (b). The arrow indicates the marked mosquito release site. Note that the compounds in the northern half of Fourda had much higher numbers of mosquitoes overall, which correlated with higher numbers of recaptures (results not shown). See text for details on sampling regime.

We give a graphic representation of all the MRR data as a trellis diagram (Figure 1). As an example of how cohorts can be followed using the trellis: on July 3, 126 mosquitoes were released of a total of 160 captured. Following the left slanting diagonal, two mosquitoes marked on July 3 were recaptured on July 4, one was recaptured on July 5, and none after that. We can also see the distribution of release dates for recaptures on any given day. For example: on March 19, three mosquitoes that had been released on March 18 were recaptured together with four from the release on March 17 and one from the release on March 16.

**Figure 2:**
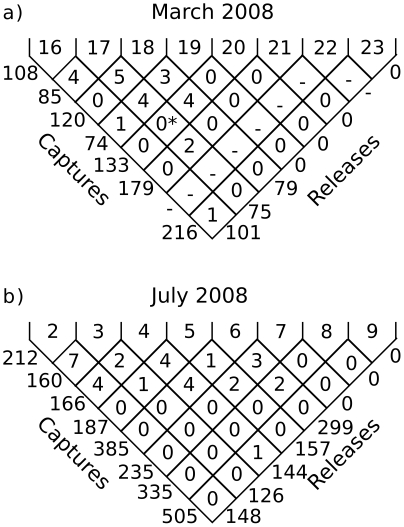
Fisher-Ford trellis plots of mark-release-recapture results obtained in Fourda. For each plot, dates are given along the top of the plot; numbers of mosquitoes marked and released for each date (follow diagonally) are shown along the right edge; numbers of mosquitoes captured are along the left edge. Numbers between these two edges represent the number of marked mosquitoes recaptured, which can be identified by cohort. See the text for further details. (a) Dry season; (b) Wet season. * Landing catch was conducted in Fourda the evening of March 20 with four collectors; four marked *An. gambiae* were recaptured, all having been released on March 17. These are not included in the analysis.

In March, we conducted extensive collections in Kenieroba: two sessions of aspiration collections in a total of 101 houses, three collections by PSC in a total of 60 houses, and night-landing catches for a total of 12 man-nights in Kenieroba to search for migrant marked mosquitoes. These collections were conducted in houses spread over 50 compounds in the southern part of the village (Figure 2) due to the very low number of mosquitoes per structure. For the July experiment, we conducted two aspiration collections in a total of 44 houses and one PSC collection in 48 houses in Kenieroba; all houses were located in four compounds nearest to Fourda along the southern edge of the village in at attempt to maximize the probability of recapturing a marked mosquito.

The extensive and multi modal collection efforts in Kenieroba yielded only 113 *An. gambiae* in March, none of which were marked. In July, we were able to capture 1994 *An. gambiae*, of which two were marked females, one released 4 days prior to capture and one 6 days.

The absolute number of males recaptured was too low to use for estimating population size in both March and July, so we have focused on the data for females. The results below thus apply to the female population in Fourda.

### Taxonomic Identification of Vectors

The majority of samples from both occasions were found to be of the M molecular form of *An. gambiae* s.s. (58.4%), with the S form making up 25.0% of the sample and hybrids of the two found in 11.5% of the samples. The S molecular form was present in proportions between 20% and about 50% relative to the M form in all village samples except in the riparian villages during July, where it was in low frequency. *Anopheles arabiensis* Patton (Diptera: Culicidae) was quite infrequent overall (5.1%) and almost always found in March.

### Estimates of Population Size

**Figure 3:**
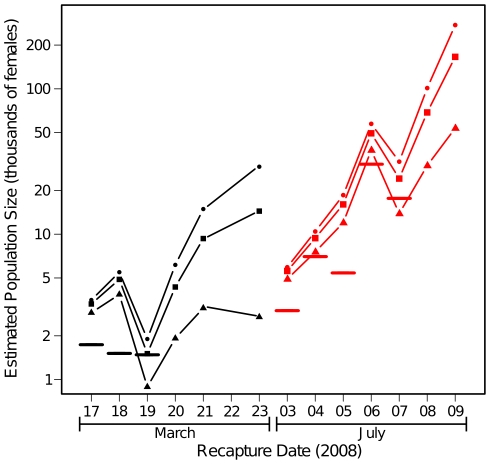
Lincoln index and Fisher-Ford estimates of population size in Fourda over time with varying survivorship. Lincoln index estimates (horizontal lines) are based on recaptures of marked mosquitoes released the previous day only. Fisher-Ford estimates per day are based on recaptures on all days prior to the estimate with an assumed daily survival rate (*s*): Triangles, *s* = 0.6; Squares, *s* = 0.8; Circles, *s* = 0.9. Black symbols and lines, dry season; Red symbols and lines, wet season.

The instantaneous estimates of population size in Fourda calculated via Lincoln Index are given in Table 2. During the dry season, these are remarkably consistent with an estimated value between 1481 and 1715 females, with a mean of 1577 and narrow confidence intervals that are consistent between dates. These estimates are also consistent with the result obtained via JHE assuming a low daily survival rate of 0.6 (estimate = 1687, 95% CI = 1191–2537; Figure 3) and with the Fisher-Ford estimates of population size during the dry season with the same low daily survival rate (mean = 2528, Figure 3).

**Table 2:** Instantaneous Population Size Estimated via Lincoln Index.a,b

Recapture date	*a*	*n*	*r*	*P*	SD(*P*)
17 March 2008	101	75	4	1535	606
18 March 2008	85	120	5	1715	632
19 March 2008	79	74	3	1481	645
03 July 2008	148	160	7	2979	968
04 July 2008	126	166	2	7014	3475
05 July 2008	144	187	4	5414	2181
06 July 2008	157	385	1	30301	17449
07 July 2008	299	235	3	17641	5700

a Lincoln index with small *r* (<20) adjustment was used.

b *P*, population size; *a*, number marked and released the previous day; *n*, number captured; *r*, number captured that were marked the previous day (recaptures).

The instantaneous estimates of population size in Fourda for the wet season are much higher and also more variable (2979 females to over 30,000, with much larger confidence intervals) with a mean of 12,670 (Table 2). These overall larger values are consistent with the JHE results, which gave estimates between about 10,500 and 30,300 (Table 3) during the wet season depending on the assumed daily survival rate, all with large confidence intervals. The Fisher-Ford method used on the July data yielded generally increasing estimates of population size, from around 5,000 females at the very start of the experiment to unrealistic values between about 50,000 and 275,000 female by the end, depending on the assumed survival rate. This is a strong indication that some assumption or assumptions of the method were increasingly divergent from reality as more time passed from the start of the experiment.

**Table 3:** Estimated population sizes for Fourda using the joint hypergeometric maximum likelihood estimator from the program NOREMARK for varying daily survival rates.

	March 2008		July 2008	
Daily Survival	Estimate	95% CI a	Estimate	95% CI a
0.6	1687	1191–2537	10507	7608–15156
0.8	3659	2556–5547	21450	15484–31015
0.9	5378	3746–8172	30293	21849–43830

a CI, confidence interval.

One important source of error that exists and may increase over time in both multiple recapture-based analyses comes from the assumed daily survivorship. We have varied daily survivorship to allow comparison of scenarios and check the sensitivity of the analysis to this factor. Clearly the daily survival rate will not be the same for all intervals, but in the absence of better information the multiple recapture results do indicate some consistency with those obtained via Lincoln index in both March and July. In this analysis, we generally see increasing values of population size estimates with increasing time since the first release (Figure 3), an indication that we are overestimating daily survivorship, or that there is a net inflow of unmarked mosquitoes or that there is an outflow of marked mosquitoes from the sampled area. These could all lead to a lower probability of re-capturing a marked mosquito than expected.

In March, we estimated the parity rate to be 0.96 from 78 unfed females captured in Fourda by night-landing catch, yielding an estimated daily survival of 0.98. In July, we fit a logarithmic decay model to the mortality data from five control cups, which gave an average estimate of daily survival of 0.80. These estimates are not easily comparable as they were derived in different ways, and additionally and utilize from different assumptions, so they are presented here for completeness but not as reliable estimates of the true daily survival rate, as discussed below.

## Discussion

We have presented results of MRR experiments that gave estimates of the population size and migration potential of the anopheline population in Fourda during the dry and wet seasons of 2008. In particular, we estimate the population size of *An. gambiae* s.s. in Fourda to be below 3,000 females during the dry season. We base this figure on the consistent and low values obtained on three different occasions using the Lincoln index (mean = 1577, mean SD = 628) and the general agreement between this value and those obtained via multiple recapture methods with low daily survivorship (JHE estimate = 1687 95% CI = 1191–2537; mean Fisher-Ford estimate = 2528).

The survivorship estimate we obtained for the dry season was very high, but it is also unreliable. Parity-based estimates of survivorship may be skewed by sampling, especially by sampling only females in the feeding phase of the life cycle as necessitated by conditions of low mosquito numbers in March [29]. In our case logistical limitations also precluded repeated longitudinal samples of parity rates, further compromising the utility of the estimate we did obtain. We suspect that the daily survivorship during the dry season is low, and perhaps even lower than survivorship during the wet season.

During the wet season, population sizes are likely much larger, though we are hesitant to state a specific figure because the wet season results require special caveats. The methods used to estimate population sizes from single or multiple marking and recapture sessions assume a closed population (not receiving migrants or producing emigrants), a condition that seems to apply to Fourda during the dry but not during the wet season. Indeed, two migrants from Fourda were found in Kenieroba in July but none in March, when the overall number of mosquitoes in Kenieroba was also found to be quite low. The relatively high recapture rate in Fourda during the dry season (10.6%) compared with the rainy season (3.5%) is primarily an indication of the higher number of mosquitoes in the area, but also suggests that the mosquitoes in Fourda in July were more likely to be connected to unsampled other areas that have isolated mosquito populations during the dry season. If this is the case, not all mosquitoes that are marked have an equal probability of being recaptured, and there may be a net inflow of unmarked mosquitoes to the area, both important deviations from the assumptions required for MRR estimates of population size. The possibility that marked mosquitoes leave the sampled area (Fourda) during the wet season and/or there is net recruitment of unmarked mosquitoes over time is supported by the massive increase in population size estimates over time in Figure 3 regardless of the assumed rate of daily survivorship.

The effect of new habitat on recruitment may be reflected in the large increase in the number of mosquitoes captured by aspiration in Fourda between July 5 and 6 (Figure 1). The number of mosquitoes captured more than doubled, indicating a significant increase in population size [30]. This is likely a consequence of recruitment from larval sites created by heavy rainfall 4–7 days earlier (data not shown). Although 4 days is insufficient for complete life cycle from egg to adult, it could be important for the maintenance of small and older breeding sites that would have dried in the absence of rain.

Our data suggest, then, that both population size and connectivity to Kenieroba increased for Fourda in July compared with March. Movement over a few kilometers has long been known for *An. gambiae* from several previous MRR studies [23], but this is not always tested between seasons (however, see [31]). One factor that is probably important in our study location is the appearance of favorable mosquito habitat and larval sites between the villages, including rainwater pools, puddles, brick pits, and increased agricultural activity. These would produce mosquitoes and provide better habitat for mosquito movement, which would foster connectivity [32] in July but not in March.

These results are consistent with a previously described model of population dynamics in this region around the Niger river [14] and may apply in other riparian regions with similar dynamics [16] as well as under a variety of analogous situations, at the scale of a single village [33] or between villages on an altitudinal gradient [34]. For our study area, the model can be described as follows: as rains diminish in November and December, larval habitats disappear and the environment becomes drier, so the population of mosquitoes in the larger inland village decreases to very few *An. gambiae*. At the same time, the Niger River retreats, leaving sandy pools in its river bed that sustain a population of mosquitoes in the thousands around the small fishing village. The riparian population persists throughout the dry season of December through April and is effectively contained to that village. Once the rains restart around May, movement of mosquitoes from the fishing village to the larger inland village allows the inland population to be seeded by a large number of migrants and grow quickly. That larger population can then be sustained by new local larval habitat created by the rains falling on mostly human-made ground cavities [14]. According to this model, at the height of the rainy season the mosquitoes in both villages might be considered to be part of the same population.

The results presented in this paper suggest that IRS or other adult mosquito targeting vector control interventions may be made more efficient by treating the low number of houses in the fishing village by the river during the dry season. Measures targeting adults are preferable to larval control due to the large number of breeding sites by the river compared to the small number of houses and the generally accepted increased efficiency of targeting the adult stage for malaria reduction [35], [36]. Doing so would decrease the size of an already small population of malaria vectors and likely would delay the explosive increase in population size recorded in the larger inland village with the rains. Ultimately, this approach could reduce the length of the effective malaria transmission season in the larger village as well as in the fishing village. We are currently monitoring entomological transmission parameters of inland-riparian village pairs where spraying was conducted by the river and in other pairs where no spraying was carried out. This study should capture effects in both inland and riparian villages of IRS interventions by the Niger.

## References

[1] Greenwood B, Mutabingwa T (2002). Malaria in 2002.. Nature.

[2] Breman JG, Alilio MS, Mills A (2004). Conquering the intolerable burden of malaria: what's new, what's needed: a summary.. Am J Trop Med Hyg.

[3] Feachem RGA, Sabot OJ (2007). Global malaria control in the 21st century: a historic but fleeting opportunity.. JAMA.

[4] Killeen GF, Seyoum A, Knols BGJ (2004). Rationalizing historical successes of malaria control in Africa in terms of mosquito resource availability management.. Am J Trop Med Hyg.

[5] Depinay J, Mbogo C, Killeen G, Knols B, Beier J (2004). A simulation model of African *Anopheles* ecology and population dynamics for the analysis of malaria transmission.. Malaria J.

[6] Carter R, Mendis K, Roberts D (2000). Spatial targeting of interventions against malaria.. Bull World Health Organ.

[7] Killeen GF, Knols BGJ, Gu W (2003). Taking malaria transmission out of the bottle: implications of mosquito dispersal for vector-control interventions.. Lancet Infect Dis.

[8] Dolo A, Camara F, Poudiougo B, Touré A, Kouriba B (2003). Epidemiology of malaria in a village of Sudanese savannah area in Mali (Bancoumana). 2. Entomo-parasitological and clinical study.. Bull Soc Pathol Exot.

[9] Warburg A, Touré Y (2002). Estivation of *Anopheles gambiae*: potential habitats and physiology..

[10] Charlwood J, Vij R, Billingsley P (2000). Dry season refugia of malaria-transmitting mosquitoes in a dry savannah zone of east Africa.. Am J Trop Med Hyg.

[11] Taylor CE, Manoukis NC (2007). Effective population size in relation to genetic modification of *Anopheles gambiae* sensu stricto.. Ecological aspects for application of genetically modified mosquitoes.

[12] Hanski IA, Gaggiotti OE (2004). Ecology, genetics and evolution of metapopulations..

[13] Dingle H (1996). Migration..

[14] Sogoba N, Doumbia S, Vounatsou P, Baber I, Keita M (2007). Monitoring of larval habitats and mosquito densities in the Sudan savanna of Mali: implications for malaria vector control.. Am J Trop Med Hyg.

[15] Omer SM, Cloudsley-Thompson JL (1970). Survival of female *Anopheles gambiae* Giles through a 9-month dry season in Sudan.. Bull World Health Organ.

[16] Jawara M, Pinder M, Drakeley C, Nwakanma D, Jallow E (2008). Dry season ecology of *Anopheles gambiae* complex mosquitoes in The Gambia.. Malaria J.

[17] Dolo A, Poudiougo B, Modiano D, Camara F, Kouriba B (2003). Epidemiology of malaria in a village of Sudanese savannah in Mali (Bancoumana). Anti-TRAP and anti-CS humoral immunity response.. Bull Soc Pathol Exot.

[18] Touré YT, Doumbo O, Toure A, Bagayoko M, Diallo M (1998). Gametocyte infectivity by direct mosquito feeds in an area of seasonal malaria transmission: implications for Bancoumana, Mali as a transmission-blocking vaccine site.. Am J Trop Med Hyg.

[19] Diuk-Wasser MA, Touré MB, Dolo G, Bagayoko M, Sogoba N (2005). Vector abundance and malaria transmission in rice-growing villages in Mali.. Am J Trop Med Hyg.

[20] Scott JA, Brogdon WG, Collins FH (1993). Identification of single specimens of the *Anopheles gambiae* complex by the polymerase chain reaction.. Am J Trop Med Hyg.

[21] Favia G, Torre AD, Bagayoko M, Lanfrancotti A, Sagnon N (1997). Molecular identification of sympatric chromosomal form of *Anopheles gambiae* and further evidence of their reproductive isolation.. Insect Mol Biol.

[22] Favia G, Lanfrancotti A, Spanos L, Sidén-Kiamos I, Louis C (2001). Molecular characterization of ribosomal DNA polymorphisms discriminating among chromosomal forms of *Anopheles gambiae* s.s.. Insect Mol Biol.

[23] Service MW (1993). Mosquito ecology: field sampling methods..

[24] Bartmann R, White G, Carpenter L, Garrott R (1987). Aerial mark-recapture estimates of confined mule deer in Pinyon-Juniper Woodland.. J Wildlife Manage.

[25] White GC (1996). NOREMARK: population estimation from mark-resighting surveys.. Wildlife Soc Bulletin.

[26] Touré YT, Dolo G, Petrarca V, Traoré SF, Bouaré M (1998). Mark-release-recapture experiments with *Anopheles gambiae* s.l. in Banambani Village, Mali, to determine population size and structure.. Med Vet Entomol.

[27] Costantini C, Li SG, Della Torre A, Sagnon N, Coluzzi M (1996). Density, survival and dispersal of *Anopheles gambiae* complex mosquitoes in a West African Sudan savanna village.. Med Vet Entomol.

[28] Detinova TS (1962). Age-grouping methods in Diptera of medical importance with special reference to some vectors of malaria.. Monogr Ser World Health Organ.

[29] Gillies MT, Wilkes TJ (1965). A study of the age-composition of populations of *Anopheles gambiae* Giles and *A. funestus* Giles in North-Eastern Tanzania.. Bull Entomol Res.

[30] Bates M (1949). The natural history of mosquitoes..

[31] Gillies MT (1961). Studies on the dispersion and survival of *Anopheles gambiae* Giles in East Africa, by means of marking and release experiments.. Bull Entomol Res.

[32] Service MW (1997). Mosquito (Diptera: Culicidae) dispersal: the long and short of it.. J Med Entomol.

[33] Ribeiro JM, Seulu F, Abose T, Kidane G, Teklehaimanot A (1996). Temporal and spatial distribution of anopheline mosquitos in an Ethiopian village: implications for malaria control strategies.. Bull World Health Organ.

[34] Kulkarni MA, Kweka E, Nyale E, Lyatuu E, Mosha FW (2006). Entomological evaluation of malaria vectors at different altitudes in Hal District, Northeastern Tanzania.. J Med Entomol.

[35] MacDonald G (1957). The epidemiology and control of malaria..

[36] Nyarango PM, Gebremeskel T, Mebrahtu G, Mufunda J, Abdulmumini U (2006). A steep decline of malaria morbidity and mortality trends in Eritrea between 2000 and 2004: the effect of combination of control methods.. Malar J.

